# Near‐term ecological forecasting for dynamic aeroconservation of migratory birds

**DOI:** 10.1111/cobi.13740

**Published:** 2021-07-05

**Authors:** Kyle G. Horton, Benjamin M. Van Doren, Heidi J. Albers, Andrew Farnsworth, Daniel Sheldon

**Affiliations:** ^1^ Department of Fish, Wildlife, and Conservation Biology Colorado State University Fort Collins Colorado USA; ^2^ Edward Grey Institute, Department of Zoology University of Oxford Oxford UK; ^3^ Cornell Lab of Ornithology Cornell University Ithaca New York USA; ^4^ Department of Economics University of Wyoming Laramie Wyoming USA; ^5^ College of Information and Computer Sciences University of Massachusetts Amherst Massachusetts USA

**Keywords:** aeroecology, bird migration, light pollution, radar, remote sensing, aeroecología, contaminación lumínica, migración de aves, radar, telemetría, 飞行生态学, 鸟类迁徙, 光污染, 雷达, 遥感

## Abstract

Near‐term ecological forecasting has the potential to mitigate negative impacts of human modifications on wildlife by directing efficient action through relevant and timely predictions. We used the U.S. avian migration system to highlight ecological forecasting applications for aeroconservation. We used millions of observations from 143 weather surveillance radars to construct and evaluate a migration forecasting system for nocturnal bird migration over the contiguous United States. We identified the number of nights of mitigation required to reduce the risk of aerial hazards to 50% of avian migrants passing a given area in spring and autumn based on dynamic forecasts of migration activity. We also investigated an alternative approach, that is, employing a fixed conservation strategy based on time windows that historically capture 50% of migratory passage. In practice, during both spring and autumn, dynamic forecasts required fewer action nights compared with fixed window selection at all locations (spring: mean of 7.3 more alert days; fall: mean of 12.8 more alert days). This pattern resulted in part from the pulsed nature of bird migration captured in the radar data, where the majority (54.3%) of birds move on 10% of a migration season's nights. Our results highlight the benefits of near‐term ecological forecasting and the potential advantages of dynamic mitigation strategies over static ones, especially in the face of increasing risks to migrating birds from light pollution, wind energy infrastructure, and collisions with structures.

## Resumen

La estimación ecológica a corto plazo tiene el potencial para mitigar los impactos negativos de las modificaciones humanas sobre la fauna al dirigir las acciones eficientes mediante predicciones relevantes y oportunas. Usamos el sistema de migración de aves de Estados Unidos para resaltar las aplicaciones de la estimación ecológica para la aeroconservación. Usamos millones de observaciones tomadas de 143 radares de vigilancia climática para construir y evaluar un sistema de estimaciones migratorias para la migración de aves nocturnas en los Estados Unidos contiguos. Identificamos el número de noches de mitigación requeridas para reducir el riesgo de peligros aéreos para el 50% de las aves migratorias que pasan por un área específica en la primavera y en el otoño con base en las estimaciones dinámicas de la actividad migratoria. También investigamos una estrategia alternativa: el uso de una estrategia fija de conservación basada en las ventanas temporales que históricamente han capturado el 50% del pasaje migratorio. En la práctica, durante la primavera y el otoño, las estimaciones dinámicas requirieron menos noches de acción en comparación con la selección de ventana fija en todas las localidades (primavera: promedio de 7.3 más días de alerta; otoño: promedio de 12.8 más días de alerta). Este patrón resultó en parte por la naturaleza pulsada de las migraciones aviarias capturadas en los datos del radar, en los cuales la mayoría de las aves (54.3%) se mueven durante el 10% de las noches durante la temporada migratoria. Nuestros resultados resaltan los beneficios que tienen las estimaciones ecológicas a corto plazo en comparación con las estáticas, especialmente de frente a los riesgos crecientes que encaran las aves migratorias por la contaminación lumínica, la infraestructura de energía eólica y las colisiones con las estructuras.

## 利用短期生态预测进行候鸟动态飞行保护

【摘要】 短期生态预测可以提供及时的相关预测来指导有效的行动, 从而减轻人类改造自然对野生动物造成的负面影响。本研究利用美国鸟类迁徙系统, 展示了生态预测在鸟类飞行保护方面的应用。我们利用 143 个气象监视雷达的数百万次观测, 构建并评估了一个针对美国境内鸟类夜间迁徙的迁徙预测系统。根据对迁徙活动的动态预测, 我们确定了鸟类迁徙所需夜晚的天数, 从而可以降低春秋季节通过特定地区 50%候鸟面临的空中危害的风险。我们还研究了另一种替代方法, 它采用基于时间窗口的固定保护策略, 可以覆盖历史上50%的迁徙通道。我们在实践中发现, 春秋两季, 基于动态预报的所有位点需要采取保护行动的夜晚数都比基于固定窗口选择所需的夜晚数要少 (春季平均相差 7.3 个警戒日, 秋季平均相差 12.8 个警戒日) 。出现这种模式的部分原因是因为雷达数据捕捉到的鸟类迁徙具有脉冲属性, 其中大多数鸟类 (53.4%) 会在一个迁徙季节 10%的夜晚中完成迁徙。我们的研究结果强调了短期生态预测的好处, 以及动态减缓策略相对于静态策略的潜在优势, 特别是在光污染、风能基础设施以及与构筑物碰撞对候鸟造成的风险日益增加的情况下。**【翻译:胡怡思;审校:聂永刚】**

## INTRODUCTION

Knowing when to direct action to protect species and habitats is essential for successful conservation (Knight et al., 2010; Wilson et al., 2005), and there are many examples of such campaigns (e.g., Burgess et al., 2019; Liberati et al., 2019; Luther et al., 2016; Wilson et al., 2019). Safeguarding highly dynamic ecological processes, such as movement and migration, poses a greater challenge (Reynolds et al., 2017). However, the spatial process of migration also creates an opportunity to reduce the amount of time during which conservation measures are necessary at any particular location. Ecological forecasting of animal movements at relevant spatial and temporal scales may provide a pathway toward real‐time conservation (Dietze et al., 2018; Van Doren & Horton, 2018). Days, hours, or even minutes can make the difference between successful intervention and missed opportunity when considering highly vagile species. Timely conservation actions relevant to migrating species may include the temporary removal of terrestrial or aquatic barriers (e.g., fences, dams), aerial obstacles (e.g., wind turbines, aircraft), or point‐source pollutants (e.g., light pollution, chemical pollution) (Marschall et al., 2011; Naidoo et al., 2012; Van Doren et al., 2017).

Among the enormous and diverse range of opportunities to safeguard migratory taxa, bird movements embody these conservation challenges, both in space and time, with movements spanning weeks to months across hundreds to thousands of kilometers through diverse ecosystems (Thorup et al., 2020; Bauer et al., 2020). Although a large percentage of migratory birds’ annual cycles may be based in terrestrial or aquatic systems, twice annually, billions of birds fill the lower atmosphere en route to wintering or breeding grounds (Dokter et al., 2018). Spring and autumn migratory seasons often encompass multiple months, but movements are not uniformly distributed in space or time (Horton et al., 2020). During any year in a given location, the majority of migrants will pass overhead within a period of days or weeks (Horton et al., 2020), but specific nights of peak migration vary across locations and years. Understanding, quantifying, and predicting this variation is essential to avian conservation.

Migratory birds increasingly encounter aerial threats from human development (Davy et al., 2017), some of which can be mitigated by specific actions. These threats are diverse in size, shape, and their impact on migratory birds. Some of these threats induce mortality directly, for instance collisions with buildings (Loss, et al., 2014), wind turbines (Loss et al., 2013), or communication towers (Gehring et al., 2009; Loss et al., 2014). Other threats are more diffuse in their impact. For example, light pollution may direct migrants to inhospitable urban spaces (Zuckerberg et al., 2016; La Sorte et al., 2017; Van Doren et al., 2017; Lao et al., 2020), putting those individuals at risk through diminished energy reserves, phenological delays, and susceptibility to predation or injury—each factor potentially resulting in difficult‐to‐measure fitness consequences. Mitigation to enhance safe passage of migrating birds is possible for some types of threats. For example, on nights of high migratory activity, lights could be dimmed or turned off on human‐made structures or activities could be changed (e.g., wind turbines stopped). Predicting the specific nights on which birds will migrate has tremendous value for safeguarding aerial passage.

A significant hurdle to implementing dynamic conservation approaches is the availability of timely alerts for when action is necessary. Remote sensing tools (e.g., radar, acoustics, infrared imaging) can measure real‐time nightly movements of avian migrants (Horton et al., 2015), providing invaluable information for conservation. But even such instantaneous measures are too late to prevent collisions. One approach to address this challenge is to leverage historical measures to identify the seasonal windows during which the majority of migration tends to occur (e.g., period that captures 50% of activity) and direct conservation action during those fixed time windows. However, migration is highly dynamic, and the timing of migratory movements is strongly influenced by shifting atmospheric conditions (Åkesson & Hedenström, 2000; Liechti, 2006; Shamoun‐Baranes et al., 2010). For this reason, migration has night‐to‐night periodicity (Åkesson & Hedenström, 2000; Deppe et al., 2015). A fixed window approach would, therefore, be apt to capture nights of both high and low migratory activity, which could lead to costly effort with limited impact and to missing important events occurring outside the fixed window. Ecological forecasts offer an alternative approach for facilitating short‐term conservation actions (Clark et al., 2001; Luo et al., 2011). Forecasts, by nature, are temporally and spatially dynamic, offering lead time for the deployment of conservation action. Van Doren and Horton (2018) built a forecasting system to predict bird migration based on radar and atmospheric conditions; however, they did not examine how to operationalize forecasts to direct conservation efforts. Analytically, this dynamic selection approach presents a modeling challenge because large movements comprise a small fraction of the duration of a migratory season (Horton et al., 2019). Although error is an inherent property of any ecological forecast, a sufficiently accurate forecast may still capture more activity across fewer nights than a historically defined window.

To address the need for conservation solutions to mitigate hazards for nocturnally migrating birds, we examined the behavior of the dynamic and fixed approaches. We quantified the utility of a near‐term forecasting system for aeroconservation (i.e., conservation of aerial habitats) with a data‐intensive approach: radar remote sensing. We asked if actions could be taken that were 100% effective in protecting birds, on how many nights would be needed to take action to protect 50% of all migratory birds passing through a given location? In the specific case of light pollution, there is evidence that immediate mitigation action can be effective (Van Doren et al., 2017, 2021). We addressed this question with a fixed window approach based on historical data and a dynamic approach based on near‐term forecasts across the continental United States.

## METHODS

### Weather surveillance radar data

We quantified nocturnal migration from 143 weather surveillance radar (WSR) stations across the contiguous United States from 1995 to 2018. We characterized the spring migratory period from March 1 to June 10 and autumn from August 1 to November 10; each season spanned a maximum of 102 nights. To capture the complete passage of migrants, radar samples were processed from sunset to sunrise at 30‐min intervals. Level‐II NEXRAD data were downloaded from the Amazon Web Services (AWS) archive (https://s3.amazonaws.com/noaa‐nexrad‐level2/index.html) and processed using the WSRLIB package (Sheldon, 2015). We identified signatures consistent with precipitation with MISTNET (Lin et al., 2019) and removed these from reflectivity factor (migration intensity) and radial velocity (migration speed and direction) measures. Although some migration may persist through periods of light precipitation, the intersection of precipitation and migratory movements tends to be mutually exclusive. Precipitation, especially heavy precipitation, halts the movement of migrants (Richardson, 1978, 1990). For reflectivity factor and radial velocity, profiles of activity were constructed from the lowest five radar scans (0.5–4.5°) at 100‐m vertical intervals from 0 to 3 km aboveground (Buler & Diehl, 2009). We extracted data from a 5‐ to 37.5‐km radius surrounding the radar. We converted reflectivity factor to reflectivity following Chilson et al. (2012), the units of which are square centimeters per cubic kilometer (i.e., *η*). We derived migrant ground speed (kilometers per hour) and direction (degrees) from velocity azimuth displays following Browning and Wexler (1968). When necessary, radial velocity was de‐aliased following Sheldon et al. (2013). We processed just over 13 million radar scans from 2115 spring nights and 2152 autumn nights.

### Migration forecast

We used the previously described profiles of activity to train seasonal bird migration forecast models. Our goal was to generate separate spring and autumn forecast models to predict migration traffic rate at 30‐min intervals, the same frequency as the radar measurements. To implement this, we used the product of radar reflectivity and groundspeed (centimeters squared per kilometer squared per hour) with a cube‐root transformation as the model's response variable. We used a gradient boosted regression tree framework (Chen & Guestrin, 2016) to capture the complex spatiotemporal interactions of migratory movements as described by Van Doren and Horton (2018). We constructed models with the XGBoost package (Chen et al., 2017) in the R environment with 13 predictors: three spatial predictors of latitude, longitude, and height above ground level (meters); two temporal predictors of ordinal date and hour after sunset; and eight atmospheric predictors of meridional wind (meters per second), zonal wind (meters per second), air temperature (degrees Celsius), surface pressure (Pascals), relative humidity (percentage), total cloud cover (percentage), visibility (meters), and mean sea level pressure (Pascals). Atmospheric predictors were extracted from the North American Regional Reanalysis (NARR) data set (Mesinger et al., 2006) and linked with radar measures to align spatially (latitude, longitude, and height aboveground) and temporally (date and hour). The NARR measures possess a spatial resolution of 32‐km, 25 hPa vertical resolution, and 3‐h temporal resolution. For variables with multiple pressure levels, we used data up to the 300 mb. We averaged weather data within 37.5 km of each radar station. We determined height aboveground by subtracting surface geopotential height from the geopotential height of each pressure level, and we linearly interpolated data at 100‐m increments from 0 to 3000 m. Temporally, we matched radar and weather data by using the weather observation closest in time to each radar observation. We trained seasonal models with the following parameters: max_depth = 12, eta = 0.01, gamma = 1, colsample_bytree = 1, min_child_weight = 5, and subsample = 0.7. The max*_*depth is the maximum depth of regression trees; eta is the step size shrinkage used in updates to prevent overfitting and to make the boosting process more conservative (0.01 is a fine‐scale update); colsample_bytree is set for subsampling of columns (no subsampling applied with a value of 1); min_child_weight is the minimum number of instances needed in each node; and subsample is the proportion of data XGBoost randomly samples from the training data prior to growing trees (Chen et al., 2017). These parameters were selected to maximize variance explained from a tuning training set that accounted for 10% of our total radar data set (see Van Doren & Horton [2018] for details).

To determine the seasonal utility of predictions produced by forecast models, models were iteratively trained with 1 year held out. For each resultant model, we made predictions of migration traffic on the held‐out year with covariates from the Global Forecast System (GFS) (https://www.ncdc.noaa.gov/data‐access/model‐data/model‐datasets/global‐forcast‐system‐gfs). We used GFS data for this exercise, rather than NARR, because GFS data offer true meteorological forecasts and represent the data source that would be used to generate real‐time bird migration forecasts. We made 30‐min predictions of migratory activity across nine years (spring 2010 to spring 2018). Predictions were aligned spatially (latitude, longitude, and height aboveground) and temporally (ordinal date, time after sunset) with radar measures derived from NEXRAD (see “Weather surveillance radar data” section). The GFS predictions have a 0.5° spatial resolution and 3‐h temporal resolution that extends 384 h (16 days) into the future. The GFS predictions are updated four times daily (00:00, 06:00, 12:00, and 18:00 UTC); however, we used only the 00:00 UTC forecast that preceded the onset of nightly migration. We constrained our analyses to these 9 years because the download of GFS data is cumbersome and requires many terabytes of storage; GFS data are archived to 2004.

### Summing nightly migration activity

We used migration night as our sampling unit; thus, we integrated our 30‐min migration activity samples from sunset to sunrise following (Horton et al., 2020). In brief, we accounted for the flow of migrants over the sampling area (i.e., WSR‐station) by multiplying *η* (defined above) by the measured groundspeed (defined above) and integrating through the night to account for the nightly passage with linear interpolation for area under the curve, resulting in centimeters squared per kilometer squared per night. We multiplied the result of the linear interpolation for area under the curve by the altitudinal resolution (0.1 km) of each profile height bin, resulting in centimeters squared per kilometer per night. We used a radar cross‐section of 11 cm^2^, which represents an average‐sized migratory species (Dokter et al., 2018; Horton et al., 2019), to yield a nightly WSR‐station traffic rate of birds per kilometer per night. We applied this procedure to measured and forecast values and used these units to summarize total passage. Because some stations had missing data in the radar archive, we used only annual radar–season combinations with at least 100 nights. During spring, this resulted in the removal of 389 radar‐year replicates (of 1119) and 467 radar‐year replicates (of 1260) during the autumn.

### Quantifying migration alerts in practice

We evaluated two approaches for directing aeroconservation action: dynamic selection and fixed window selection. To compare these approaches, we used as a reference the number of nights needed to capture 50% of migratory activity. Under the dynamic selection scenario, we identified the minimum number of nights of conservation action (hereafter action nights) needed to capture 50% of seasonal activity. We applied dynamic selection in two ways. First, we identified nights based on the realized migration passage measured by the radars, as if we could predict the truth with complete accuracy (hereafter idealized dynamic action nights). Second, we identified nights based on our migration forecast, which is imperfect (hereafter forecast dynamic action nights). In practice, action nights would be triggered by a threshold of activity, meaning nights below the threshold receive no action and those above receive action. Thresholds are expected to vary across our coverage area.

We computed the number of forecast dynamic action nights to capture different quantiles of migration activity as follows. First, we predicted the migration intensity for each night in the held‐out year with a seasonal model trained on the remaining years. Then, for each quantile ranging from 0.05 to 0.95 by increments of 0.05, we searched for the smallest threshold of migration activity (*t*) such that the nights with predicted intensity greater than or equal to *t* captured at least the desired fraction of total seasonal migration. For example, we defined the threshold at the 90^th^ percentile of activity for a WSR‐station and subsequently determined how many forecast nights per season were captured as action nights. For those nights labeled as action nights, we also determined the percent activity (from known historical measures) captured in those events (e.g., the 90^th^ percentile results in 10 action nights that capture 50% of activity). We searched for thresholds with predicted migration intensities rather than measured ones because intensities were not perfectly calibrated in terms of magnitude, thus we opted to use thresholds from predicted values to account for differences (Van Doren & Horton, 2018). We defined the threshold from forecast predictions from all years except the year of interest.

The fixed window selection approach identified a minimum continuous window of time that historically captures 50% of migration activity. This approach did not rely on ecological forecasting and was seasonally fixed but spatially variable. To quantify the optimal seasonal window of time for each WSR‐station, we iterated through window widths ranging from 1 to 100 nights and stepped through each combination of window width and start time (e.g., a window of 10 nights starting on April 15). For each combination, we examined the percentage of activity captured on an annual basis. We averaged the percent capture across all years and selected the optimal window that minimized duration but captured at least 50% of migratory activity. For determining the efficacy of this approach in practice, we held out the year of interest when determining the optimal window.

## RESULTS

### Passage metrics from idealized dynamic and fixed window selection

**FIGURE 1:**
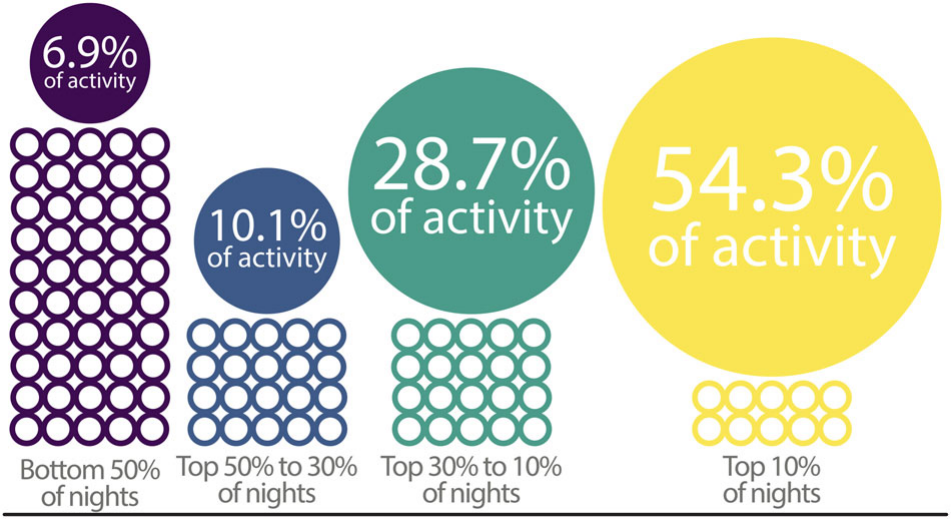
Mean percentage of nighttime migration activity captured across 4 percentile categories clear from figure (open circles, number of nights per season in each category; large, solid circles, migration activity scaled by summed percentage of activity in each category and average of spring and autumn seasons across all weather surveillance radar stations)

**FIGURE 2:**
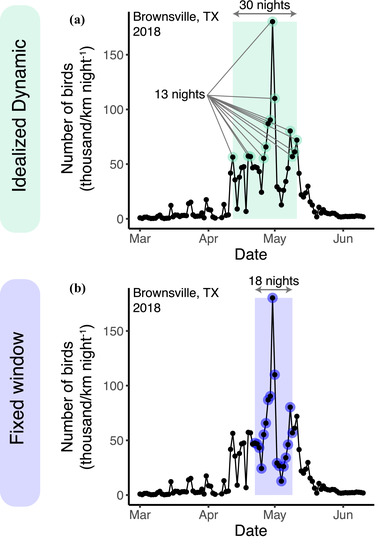
Scenarios for selecting nights for conservation action for Brownsville, Texas (USA) during spring 2018 migration for (a) idealized dynamic selection (13 nights that capture 50.5% of total passage across a window of 30 nights) and (b) fixed window selection (historically defined window of peak activity and for 2018, 52.1% of migration activity)

**FIGURE 3:**
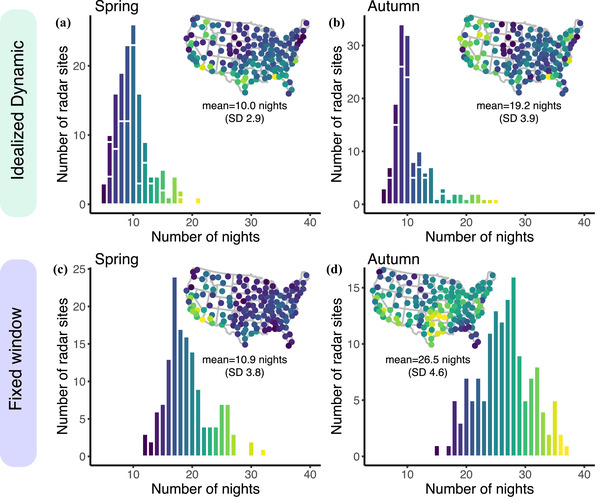
Mean number of nights required to dynamically capture 50% of migration activity in an idealized setting in (a) spring and (b) autumn and (c) spring and (d) autumn mean fixed window that historically captures 50% of migration activity (color scales vary)

Across 1628 unique sampling nights (92,296 spring and 85,315 fall nightly samples), the majority of total migratory passage (54.3%) occurred on 10% of nights for each season (Figure 1). Under idealized dynamic selection (Figure 2a), 10.0 nights (SD 2.9) during the spring (Figure 3a) and 10.9 nights (SD 3.8) during autumn (Figure 3b) captured 50% of activity at each station. These nights occurred in a continuous span of 34.7 nights (SD 9.8) during spring and 48.4 nights (SD 10.0) during autumn. In both seasons, the majority of migration occurred on fewer nights farther north (linear model showing effect of latitude: spring, −0.27 [SD 0.07], *p* < 0.001; autumn, −0.18 [SD 0.09], *p* < 0.001) and farther east (linear model showing effect of longitude: spring, −0.05 [SD 0.03], *p* = 0.002; autumn, −0.14 [SD 0.04], *p* < 0.001).

Fixed windows that captured 50% of passage (Figure 2b) spanned 19.2 nights (SD 3.9) in spring (Figure 3c) and 26.5 nights (SD 4.6) in autumn (Figure 3d). Window width generally decreased farther north (linear model, spring, −0.08 [Confidence Interval (CI) 0.11], *p* = 0.159; autumn, −0.19 [CI 0.13], *p* = 0.005) and farther east (linear model, spring, −0.08 [CI 0.04], *p* < 0.001; autumn, −0.01 [CI 0.05], *p* = 0.728). However, these linear spatial dependencies were weaker than the idealized dynamic selection trends and at times not significant. The fixed window selection approach required significantly more time than idealized dynamic selection to capture 50% of activity (paired *t*‐test, spring mean of differences 9.3 nights, *t*_142_ = –36.5, *p* < 0.001; autumn mean of differences 15.6 nights, *t*_142_ = −41.7, *p* < 0.001). In both idealized dynamic and fixed window scenarios, spring periods were significantly shorter than autumn periods (paired *t*‐test, mean dynamic seasonal difference 1.0 nights, *t*_142_ = −3.2, *p* = 0.002; mean fixed‐window seasonal difference 7.2 nights, *t*_142_ = −15.1, *p* < 0.001).

### Forecast passage metrics

On average, our forecast models based on NARR reanalysis data explained 73.0% (SD 0.008) of the variance of the cube‐root‐transformed migration intensity during spring and 69.8% (SD 0.010) during autumn. Using the Global Forecast System to predict migration traffic 1 day in advance, our spring model explained 70.4% (SD 0.009) of the variance and 68.8% (SD 0.009) of the variance in autumn.

**FIGURE 4:**
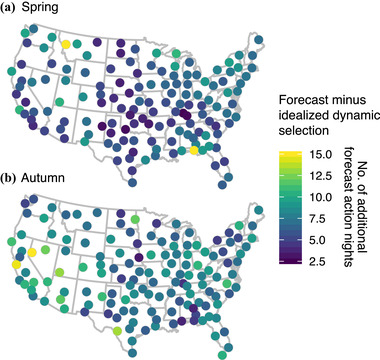
Differences between number of action nights between forecast and idealized dynamic selection approaches for (a) spring and (b) autumn migration. The number of action nights for both methods is that needed to capture 50% of activity

**FIGURE 5:**
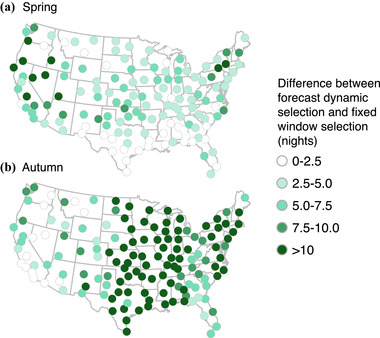
Differences between number of action nights in forecast dynamic selection and fixed window selection in (a) spring and (b) autumn. The number of action nights for both methods is that needed to capture 50% of migration activity. In all cases, fixed window required more nights than forecast dynamic

Because migration forecasts are imperfect, more action nights were required to capture 50% of migration activity compared with an idealized scenario (above; Figure 4). During spring, 13.7 (SD 3.5) forecast dynamic action nights were necessary and 15.9 (SD 4.6) during autumn. However, this was still far fewer than with fixed selection, which required 53% more action nights in the spring (mean of 7.3 more alert days) (Figure 5a) and 81% more action nights in autumn (mean of 12.8 more alert days) (Figure 5b). At all WSR stations, forecast dynamic selection resulted in fewer action nights needed to capture 50% of migratory passage compared with fixed window selection (Figure 5).

**FIGURE 6:**
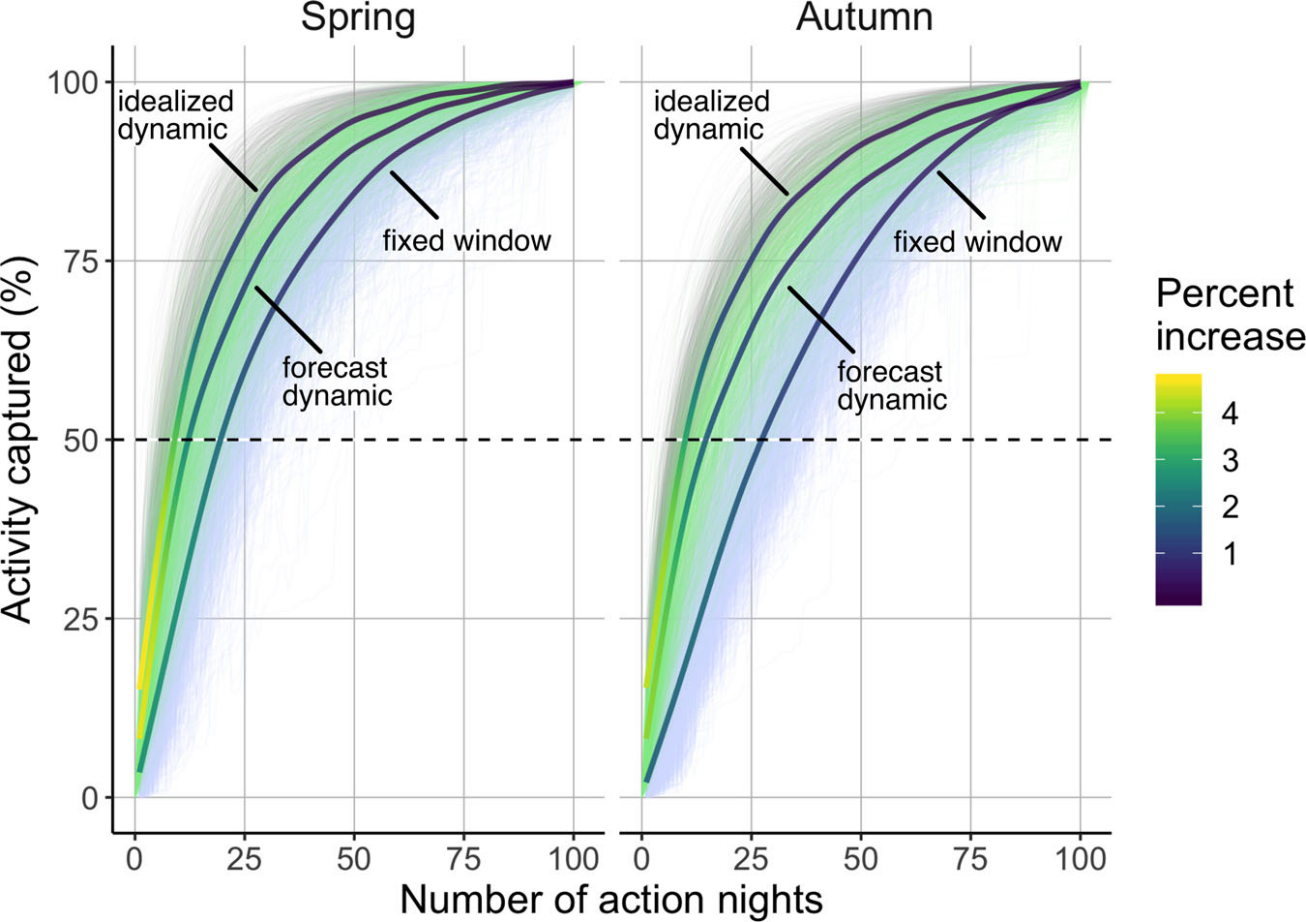
Relationship between number of action nights and migration activity captured for idealized dynamic, forecast dynamic, and fixed window selection (i.e., gray, green, and blue, respectively) in spring and autumn (lines, annual cumulative migration traffic rates for individual weather surveillance radar stations from spring of 2010 to spring of 2018). Each method is fitted with a generalized additive model, and the line shading signifies the rate of increase in percent activity captured

We used a benchmark of capturing 50% of migratory activity. We also examined the continuous gradient of migratory activity and number of action nights across the idealized dynamic, forecast dynamic, and fixed window selection approaches (Figure 6). Consistently across our sampling space, forecast dynamic selection captured more activity with fewer action nights than fixed window selection. Generally, after capturing 75% of migratory activity, the percent gain for each additional action night began to taper off (Figure 6).

## DISCUSSION

At present, conservation action often embodies a tension between society's desire to protect species and society's willingness to incur costs for that protection (Miller & Hobbs, 2002; Singh et al., 2015). In the era of big data, one can design strategies that provide conservation benefits at less cost—here identifying fewer action nights—to reduce this tension. We found that near‐term ecological forecasting aided realization of dynamic and optimized action. Such forecasting can perform more efficiently than status‐quo techniques and create a path for dynamic, real‐time conservation alerts that reduce society's costs of conservation. At all locations we examined, forecasting resulted in fewer action nights than static, fixed window approaches that captured comparable aerial passage and alerted protective actions.

We defined two important criteria: number of action nights as a proxy for costs and our policy goal of capturing 50% of migration passage as a proxy for an important ecological benchmark. We identified a set of dates for a fixed time window and for forecast dynamic mitigation approaches that can have the greatest impact per cost incurred. This approach does not capture all costs, including opportunity costs, of each action night and does not capture all benefits of migratory bird conservation. Instead, this approach sets the ecological goal of 50% of migration captured and asks how to minimize the action nights (costs) to achieve that goal. This cost‐effectiveness approach avoids the complications of determining the socially preferred level of conservation for economic efficiency that requires a full assessment of all market and nonmarket costs and benefits. Using action nights as a proxy for costs corresponds to the reserve site‐selection literature's use of the number of sites as a cost proxy and minimization of the number of sites chosen for a reserve network that conserves a specific number of species. That process matches only the cost‐minimizing reserve network to achieve a level of species conservation if all land units have the same cost (Ando et al., 1998; Polasky et al., 2008). It may be possible to find a set of sites that provides the target level of conserved species for a lower cost than in the site‐minimizing reserve. Here, if costs are heterogenous across nights, economic cost‐effectiveness shifts action nights toward less costly nights, which can mean more action nights but lower cost overall. One potential next step to improving the cost‐effectiveness of dynamic mitigation involves assessing the heterogeneity of action‐night costs to take advantage of opportunities to provide collision mitigation at a lower cost.

Incorporating other economic considerations could further increase conservation per dollar through appropriate use of near‐term forecasting information. First, positive correlations between higher cost action nights and numbers of migrating birds make conservation more expensive, whereas negative correlations create efficiency gain opportunities (e.g., Figge, 2004; Koellner & Schmitz, 2006; Schindler et al., 2010). For example, if nights with high wind pose a high opportunity cost of energy generation by turning off wind turbines but high wind also prevents many birds from migrating, the daily heterogeneity in costs can be leveraged to achieve the mitigation goal at lower cost (Hayes et al., 2019). Second, cost‐effectiveness relies on the characteristics of the dynamic versus fixed window approaches’ cost functions and the differences between these cost functions. Each approach's cost function likely contains a fixed cost (e.g., costs incurred to lay the groundwork to use action nights) and variable costs (e.g., costs incurred as a function of the number of action nights). Assessing the relative impact of the fixed and variable costs across the fixed window and dynamic action night choices could identify situations in which the dynamic action nights approach provides particularly large or small cost improvements over the fixed window approach. Similarly, both fixed window and forecast dynamic conservation costs for avian conservation might include costs of the foregone energy generation of turning off wind turbines (Kennedy, 2005; Cullen, 2013), which interacts with energy source‐switching costs (Bird et al., 2016) or the costs of turning off lights in urban or energy‐generation sites. Third, dynamic conservation may provide information that engages individuals in a positive way, which could create a social benefit that reduces the action night's social costs. Further economic efficiency analysis that addresses the specific costs of fixed window and dynamic conservation approaches, the heterogeneity of costs across space and time, and the engagement of potential participants could further improve the efficiency of conservation action decisions and provide the target level of conservation at a lower cost.

Although our forecasting approach already shows improvements over static approaches, at least in terms of reducing the number of action nights, we predict that the efficiency and accuracy of this dynamic approach will continue to improve with each passing migration season through the addition of new training data, inclusion of commentary sensors, and advances in computational machinery. Methodologically, we believe our predictions will improve through additions of landscape variables (e.g., land cover, greenness), finer temporal updates (e.g., every 3 h), broader spatial predictors of synoptic weather conditions, and the integration of within‐season migration activity measurements. Furthermore, we expect the explicit integration of natural history data (e.g., species observations) will enhance taxonomic resolution, increase the specificity of conservation decision‐making, and reveal potential biases of our approach, particularly in light of stratified timing of migrant passage either by species or higher taxonomic classification (Horton, Van Doren, et al., 2019). Although our threshold of protecting 50% activity is a subjective choice, our approach is extensible to conservation or economic priorities that may dictate different levels of protection (see Figure 4 for data on 25% and 75% thresholds).

Spatial heterogeneity exists in the geographic distribution of action nights in spring and autumn. For example, California and the Desert Southwest required larger numbers of action nights for both idealized dynamic and fixed window selection relative to the rest of the United States, reflecting more protracted migration passage through those regions (Figure 3). Additional anomalies during spring were evident in Texas and portions of the southeast. Although forecast dynamic selection yielded fewer action nights than fixed window selection, deviations between forecast and idealized dynamic selection were still high in some regions (Figure 4). It is likely that the complexities of topographic features, such as coastlines and terrain (e.g., Rocky Mountains), are not sufficiently captured by our model and highlight the challenge of forecasting movements in these regions. Additionally, differences between forecast and idealized selection were higher in autumn than spring. Variability of autumn movements may be larger due to age‐specific departure and flight strategies (Mitchell et al., 2015) and elevated selection of weather events to promote southward flights (Horton et al., 2016), manifesting in large flights over a wider range of time (Figure 3). Capturing these spatial patterns is important from a conservation standpoint and in the context of economic cost‐effectiveness, wherein action nights may have different inherent values.

We have demonstrated that near‐term ecological forecasting can address conservation challenges that evolve rapidly in space and time. Our approach uses volumes of data gathered to learn associations of avian migration and atmospheric conditions (Van Doren & Horton, 2018). We believe these tools, both in forecasting and alerting, can be translated directly to areas with existing radar infrastructure and archives. These approaches may encompass whole continents (e.g., Europe, Asia, or Australia), but are applicable at smaller spatial scales, requiring only a small number of radar installations. Big data analytics have arrived, particularly in wildlife ecology through large data collection efforts founded on sensor networks (e.g., radar, community science). These applications reinforce the power of these growing repositories for building new and better performing forecasts. Ecological forecasting lends itself to many conservation challenges across a wide variety of taxa and scales. For instance, predicting the emergence of ephemeral insects blooms (Stepanian et al., 2020), nesting returns of sea turtles (Van Houtan & Halley, 2011), and movements of terrestrial migrants through fragmented and shifting landscapes (Fischer & Lindenmayer, 2007; Lendrum et al., 2013; Geremia et al., 2020). Each of these examples is linked integrally with shifting climate, seasonal weather, and landscape and oceanic variability, requiring models that adapt to current conditions. Rethinking conservation goals in this dynamic framework opens new opportunities in the face of the growing intersection between humans and wildlife.

## Supporting information

Figure S1: (A) Spring and (B) autumn differences between number of action nights between forecasted and idealized dynamic selection.
