# Supplementary material for: Near‐term ecological forecasting for dynamic aeroconservation of migratory birds
Source: Conserv Biol. 2021 Jul 5;35(6):1777–86. doi: 10.1111/cobi.13740 (PMC9290813; doi:10.1111/cobi.13740)
Supplement: Supplementary file 1 — Figure S1: (A) Spring and (B) autumn differences between number of action nights between forecasted and idealized dynamic selection. [file COBI-35-1777-s001.docx]

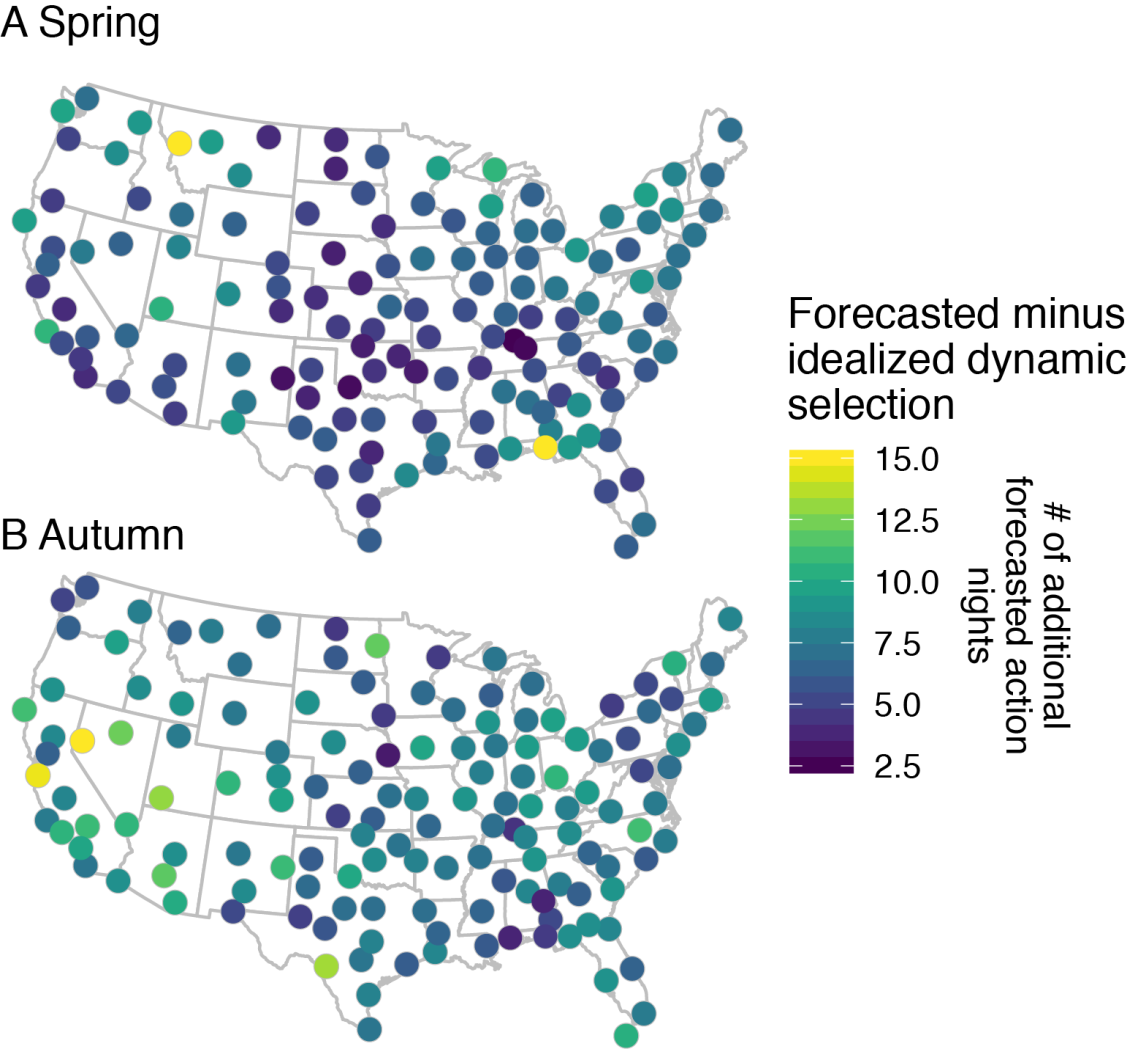


**Figure S1:** (A) Spring and (B) autumn differences between number of action nights between forecasted and idealized dynamic selection. The number of action nights for both methods is that needed to capture 50% of activity.
